# Counteraction of Tetherin Antiviral Activity by Two Closely Related SIVs Differing by the Presence of a Vpu Gene

**DOI:** 10.1371/journal.pone.0035411

**Published:** 2012-04-17

**Authors:** Kristina Nikovics, Marie-Christine Dazza, Michel Ekwalanga, Fabrizio Mammano, François Clavel, Sentob Saragosti

**Affiliations:** 1 INSERM U941, Paris, France; 2 Univ Paris Diderot, Sorbonne Paris Cité, IUH, Paris, France; 3 Laboratoire des Cliniques Universitaires, Université de Lubumbashi, Lubumbashi, Democratic Republic of Congo; Lady Davis Institute for Medical Research, Canada

## Abstract

In different primate lentiviruses, three proteins (Vpu, Env and Nef) have been shown to have anti-tetherin activities. SIVden is a primate lentivirus harbored by a *Cercopithecus denti* (*C. denti*) whose genome code for a Vpu gene. We have compared the activity of HIV-1 Vpu and of SIVden Vpu on tetherin proteins from humans, from *C. denti* and from *Cercopithecus neglectus* (*C. neglectus*), a monkey species that is naturally infected by SIVdeb, a virus closely related to SIVden but which does not encode a Vpu protein. Here, we demonstrate that SIVden Vpu, is active against *C. denti* tetherin, but not against human tetherin. Interestingly, *C. neglectus* tetherin was more sensitive to SIVden Vpu than to HIV-1 Vpu. We also identify residues in the tetherin transmembrane domains that are responsible for the species-specific Vpu effect. Simultaneous mutation (P40L and T45I) of human tetherin conferred sensitivity to SIVden Vpu, while abolishing its sensitivity to HIV-1 Vpu. We next analyzed the anti-tetherin activity of the Nef proteins from HIV-1, SIVden and SIVdeb. All three Nef proteins were unable to rescue virus release in the presence of human or *C. denti* tetherin. Conversely, SIVdeb Nef enhanced virus release in the presence of *C. neglectus* tetherin, suggesting that SIVdeb relies on Nef in its natural host. Finally, while HIV-1 Vpu not only removed human tetherin from the cell surface but also directed it for degradation, SIVden Vpu only induced the redistribution of both *C. denti* and *C. neglectus* tetherins, resulting in a predominantly perinuclear localization.

## Introduction

Evolution of mammalian species, and of primate species in particular, has been strongly affected by retroviral infections [1], [2], [3], [4], [5], [6]. In defense against retroviruses several species have evolved cellular protein that can protect cells against retroviral infection, termed restriction factors. One recently identified restriction factor, tetherin/BST2/CD317/HM1.24, is a membrane protein that exerts its antiviral effect by retaining retroviral particles (but also virions of other enveloped viruses) at the surface of infected cells, thereby strongly reducing viral propagation [7], [8], [9], [10], [11], [12]. In a number of cell types, tetherin expression is induced by type I interferon, and can therefore be considered as one component of innate immunity [11], [13]. In turn, some retroviruses have evolved a variety of countermeasures that aim at reducing the level of surface expression of tetherin in infected cells.

Primate lentiviruses principally use two viral proteins, Nef and Vpu, to down-regulate surface expression of tetherin [11], [13], [14], [15], [16], [17], [18], [19]. The extent to which downregulation of tetherin is the consequence of an activity of either of these two proteins varies widely across primate lentiviral lineages, essentially as a function of the ability of tetherins from the natural host species of these viruses to respond to either Nef or Vpu. In viruses that do not express Vpu, Nef usually mediates anti-tetherin activity. This is the case for the majority of lentiviruses from non-hominid primates, best illustrated by the strong ability of Nef from SIVmac to counteract tetherin from Rhesus macaques [16], [18], [19], [20]. In contrast, Vpu is the only protein able to down-regulate tetherin in HIV-1, a property explained by the fact that human tetherin carries a 5 amino acid deletion in its intracytoplasmic domain that makes it insensitive to regulation by Nef [3], [16].

Between these two extremes, a few viral lineages express both Nef and Vpu. Some of these viruses, such as SIVcpz, a group of chimpanzee viruses from which HIV-1 was transmitted to humans, rely essentially on Nef to counteract tetherin expression, in spite of the presence of a Vpu protein [6], [18], [21]. In other viruses, such as SIVmus, regulation of tetherin is achieved by Vpu rather than by Nef [18]. The reason why these viruses rely on Vpu rather than on Nef for regulation of tetherin, in spite of the fact that the tetherin protein of their natural hosts does not bear a deletion in their intracytoplasmic domain, remains unclear. One possible explanation for relying either on Nef or Vpu for regulation of tetherin is that a number of primate lentiviruses may have been recently transmitted to their host species and that their Vpu protein may not be optimized for regulation of the tetherin protein expressed in the new host. Some extent of species-specificity has been described for the regulatory effect of Vpu proteins [17], [18], [22]. Furthermore, the ability of some HIV-1 Vpu proteins to antagonize macaque tetherin *in vitro* and *in vivo* was reported recently [23].

To gain further insight into primate lentiviral regulation of tetherin proteins, we have focused our attention on two closely related monkey viruses, SIVden from *C. denti*, which expresses a Vpu protein [24], and SIVdeb from *C. neglectus*, a virus that belongs to a lineage phylogenetically close to SIVden but that does not express a Vpu protein [24], [25]. We initially compared the sensitivity of human, *C. denti* and *C. neglectus* tetherins to SIVden Vpu and HIV-1 Vpu. Counteraction of tetherin antiviral activity showed a species-specific pattern, and some key residues in Vpu involved in antagonizing BST2 were identified by mutagenesis. We then analyzed the effect of Nef proteins from HIV-1, SIVden and SIVdeb on tetherin from the three host species. Significant counteraction of tetherin activity was observed only when Nef from SIVdeb (which does not encode a vpu gene) was used against tetherin from its natural host, C neglectus. These findings are discussed from the perspective of the evolutionary forces acting on the viruses and their hosts.

## Results

### Comparison and expression of human, *C. denti* and *C. neglectus* tetherin proteins

**Figure 1:**
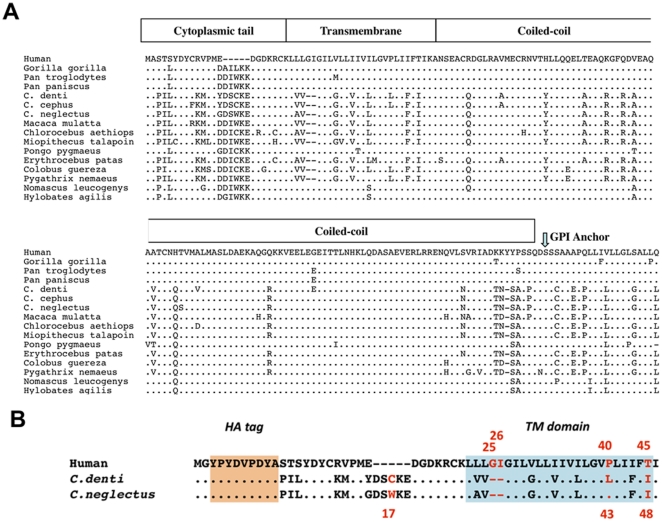
Alignment of tetherin amino acid sequences. A. Alignment of the cytoplasmic tail and transmembrane domains of tetherins from 16 primates. The accession numbers of the sequences used in this figure are: Human NP 004326; *Gorilla gorilla* ADI58594; *P. troglodytes* ADI58593; *P. paniscus* ADI58595; *C. nictitans* ACX46125; *C. mona* ACX46126; *C. cephus* ACX46508; *P. nemaeus* ADI58604; *C. aethiops* ADI58600; *E. patas* ADI58599; *P. pygmaeus* ADI58596; *M. mulatta* ADI58602; *N. leucogenys* ADI58597; *H. agilis* ADI58598; *C. guereza kikuyuensis* ADI58603; *M. talapoin* ADI58601. B. Differences in the TM domain of human, *C. denti* and *C. neglectus* tetherins are highlighted in the blue box. Red numbers and letters indicate the amino acids we mutated in this study. In both panels, amino acid identity is indicated by dots and sequence gaps are indicated by dashes.

As shown on figure 1, alignment of tetherin amino acid sequences from humans, *C. denti* and *C. neglectus* reveals a number of important differences. The first such difference is the deletion of 5 amino acids in the intracytoplasmic domain of human tetherin, the cause of its insensitivity to Nef proteins [3], [16]. In addition, several notable differences (8 amino acid substitutions and an insertion of two amino-acids) are also seen between the three tetherin proteins in the transmembrane (TM) domain, a region known to command susceptibility to down-regulation of tetherin by HIV-1 Vpu [13], [16], [26], [27], [28], [29]. These differences in both the cytoplasmic and transmembrane regions of tetherin are indicative of strong potential differences in their susceptibility to down-regulation by Vpu and Nef proteins.

**Figure 2:**
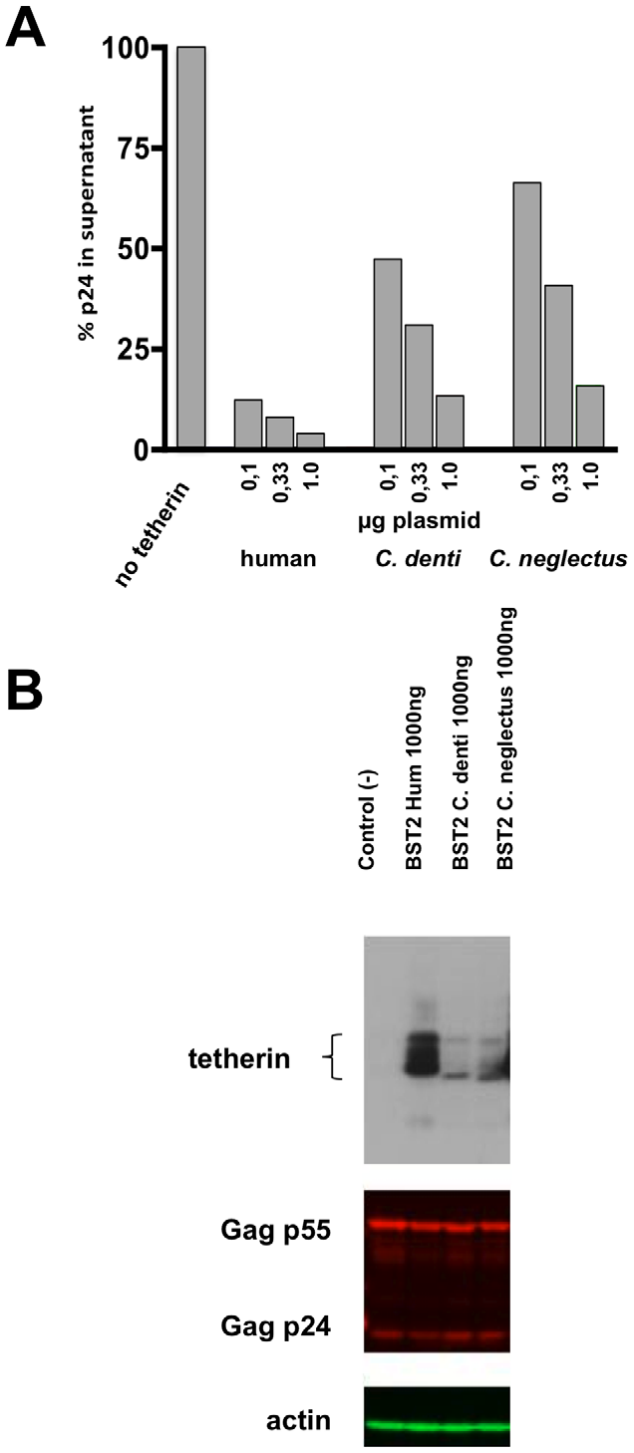
Effect of tetherin from different hosts on virus release. A. Virus particle release was tested in 293T cells transfected with HIV-1ΔVpuΔNef NL4.3 proviral DNA (500 ng) in the presence of human, *C. denti* and *C. neglectus* tetherin. Virus production expressed as a percentage of maximal particle release in the absence of tetherin is shown for increasing amounts of plasmid DNA encoding for the three tetherin constructs (100, 330 and 1000 ng). Differences in the amount of plasmid DNA in each transfection were compensated by the addition of control vector (pcDNA3). After 42 h, the amount of virus released into cell culture supernatant was measured by HIV-1 p24 ELISA. This figure is representative of three independent experiments. B. Expression of Vpu constructs. Western blot analysis was performed to determine the expression levels of the three plasmids encoding HA-tagged tetherins. 293T cells were cotransfected with 1000 ng of each plasmid. Tetherin migrated as several species in SDS-PAGE analyses, presumably as a result of heterogeneus glycosylation. Forty-two hours after transfection cell lysates were collected and analyzed by Western blot. The expression level of HIV-1 Gag protein was monitored with an anti-p24 antibody. Actin was used as a loading control.

We generated plasmids expressing human, *C. denti* and *C. neglectus* tetherin sequences tagged with a hemagglutinin (HA) epitope at their N-terminus. Human epithelial 293T cells, a cell line that does not express human tetherin unless treated with type I interferon [11], [13], were co-transfected with one of the three tetherin expression plasmids and with an indicator HIV-1 genome able to assemble and release HIV-1 virions but that does not express Vpu or Nef (pNL4.3-ΔVpuΔNef). Tetherin activity was expressed as the percentage of HIV-1 particles released in the supernatant, based on HIV-1 p24 Elisa, relative to the amount of particles released in the absence of transfected tetherin expression plasmid. Because the readout of our experiments was the ability of tetherin proteins to inhibit viral particle release, we chose to normalize the amounts of transfected tetherin expression plasmids based on this antiviral activity. To determine how much of each of the three plasmids was to be transfected to achieve comparable antiviral activity, three serial dilutions (1, 0.3 and 0.1 micrograms) of the plasmids were transfected in 293T cells together with the indicator pNL4.3-ΔVpuΔNef genome. As shown on figure 2A, inhibition of virion release by human tetherin was much stronger than that obtained with *C. denti* tetherin or of *C. neglectus* tetherin, a phenomenon essentially explained by the higher level of stable expression of human tetherin in 293T cells, as it can be seen in figure 2B. Transfection of 1 microgram of *C. denti* tetherin or of *C. neglectus* tetherin expression plasmid reduced pNL4.3-ΔVpuΔNef virion release to levels that represented 10–15% of those obtained in absence of tetherin. A comparable level of inhibition was measured when only 0.1 microgram of human tetherin expression plasmid was transfected (figure 2A). Therefore, we chose to use these amounts of the respective plasmids, yielding comparable levels of tetherin antiviral activity, in all further experiments.

### Species-specificity of tetherin antagonism by HIV-1 and SIVden Vpu

Susceptibility of each of the three tetherin proteins to antagonism by different Vpu proteins was evaluated by co-transfecting 293T cells with normalized amounts of tetherin expression plasmid, together with the indicator pNL4.3-ΔVpuΔNef genome and a plasmid expressing the Vpu protein of HIV-1 or SIVden.

**Figure 3:**
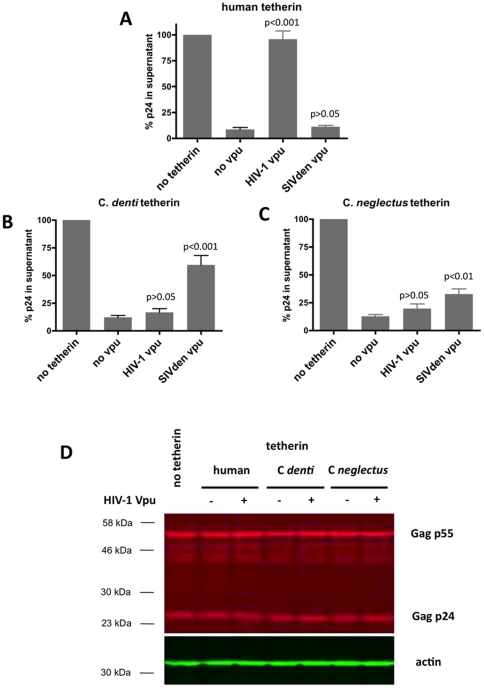
Effect of Vpu from HIV-1 and SIVden on virus release in the presence of the different tetherin molecules. Viral particle release from 293T cells was measured after cotransfection of a HIV-1ΔVpuΔNef proviral construct (500 ng) with different tetherin-encoding plasmids: (A) human (100 ng), (B) *C. denti* (1000 ng), and (C) *C. neglectus* (1000 ng), and in the absence or presence of HIV-1 or SIVden Vpu encoding plasmids (250 ng). (A, B and C). Viral particle release was quantified 24 h post-transfection by p24 ELISA. The amount of p24 antigen produced relative to the virus yield obtained in the absence of tetherin (100%) is plotted. Results are expressed as mean ± SD for 5 experiments. The indicated p values correspond to the comparison with the “no vpu” sample. (D) Forty-two hours after transfection cell lysates were collected and analyzed by Western blot. The expression level of HIV-1 Gag protein was monitored with an anti-p24 antibody. Actin was used as a loading control. The depicted gel is representative of three independent experiments. For all viral release assays, the cell-associated p24 was monitored. Molecular weight markers are shown on the left in kilodaltons.

With human tetherin, only HIV-1 Vpu was found to strongly antagonize the antiviral effect of tetherin (figure 3A), contrasting with SIVden Vpu, which failed to exert any antagonistic effect.

With *C. denti* tetherin, the only significant antagonistic effect was seen with SIVden Vpu, for which the rescue of particle release reached 60% of that of pNL4.3-ΔVpuΔNef expressed without tetherin (figure 3B). Mirroring the lack of effect of SIVden Vpu on human tetherin, HIV-1 Vpu had no significant antagonistic effect on *C. denti* tetherin. The tetherin protein of *C. neglectus* a monkey species naturally infected by a virus that does not express a Vpu protein, was moderately but significantly susceptible to antagonism by SIVden Vpu, but not susceptible to HIV-1 Vpu (figure 3C). These findings corroborate that the antagonistic properties of the Vpu protein from a given primate lentivirus are for the most part specific for the tetherin expressed by its natural host species, a sign of strong species adaptation of these viruses [17], [18], [22].

In all of these experiments, we ascertained that the changes in HIV-1 p24 content of culture supernatants that we observed were actually related to changes in virion release: as shown on figure 3D, neither tetherin nor Vpu expression had any effect on overall expression of HIV-1 Gag in transfected cells.

Although the species-specificity of tetherin antagonism by Vpu is known to be determined by sequences in the transmembrane region of tetherin, the role of particular residues in this region remains unclear. Comparison of the transmembrane domains of *C. denti* and *C. neglectus* tetherins revealed only two differences at positions 28 (V vs. A) and 43 (L vs. P) in *C. denti* tetherin numbering. Comparison of the transmembrane domains of human and *C. denti* tetherins revealed eight differences at positions 23, 24, 30, 33, 36, 40 and 45 (in human tetherin numbering) and 2 amino acid indel positions 25–26.

**Figure 4:**
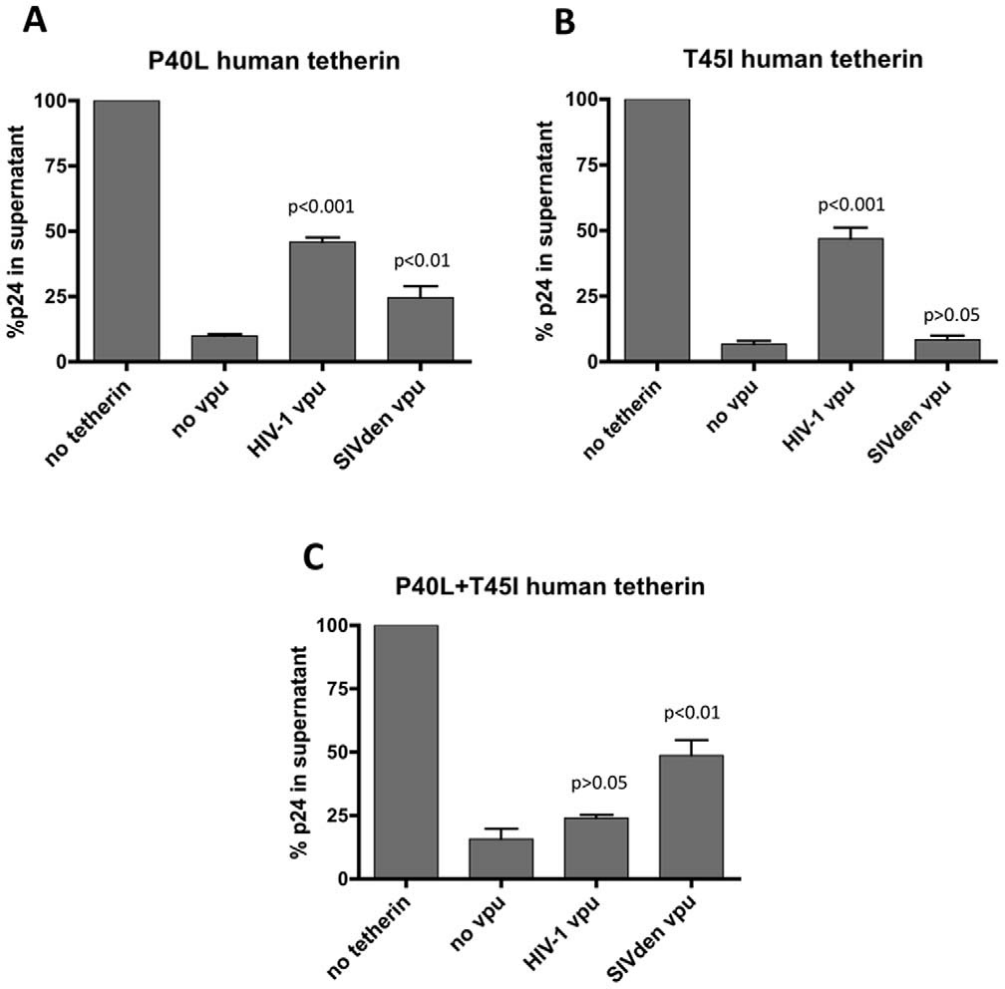
Virion release from cells tranfected with vectors expressing tetherin mutants. HIV-1 Vpu and SIVden Vpu were tested for their ability to rescue p24 release for HIV-1ΔVpuΔNef in 293T cells expressing human tetherin delta 25–26 (A) or *C. denti* tetherin insertion 28–29 (B). Twenty-four h after transfection the p24 content of the supernatant from each sample was quantified using a p24 ELISA. and expressed as the percent of p24 of the control without tetherin. Results are the mean ± SD for 5 independent experiments. The indicated p values correspond to the comparison with the «no vpu» sample.

It was reported that amino acid sequences in the transmembrane domain of human tetherin is critical for the interaction with HIV-1 Vpu but the molecular basis behind the Vpu counteraction of tetherin is still not fully elucidated. An important difference between human and *C. denti*/*C. neglectus* tetherins is the presence of a 2 amino acid insertion (glycine-isoleucine) in human tetherin at positions 25–26. These 2 amino acid are deleted in tetherins proteins from Old World monkeys, but presents in hominoids and New World Monkeys [3]. Deletion of these two amino acid in human tetherin only produced an overall drop in HIV-1 Vpu susceptibility with no increase in SIVden Vpu antagonism (figure 4A). Similarly, insertion of these two amino-acids in *C. denti* tetherin produced a moderate drop in susceptibility to SIVden Vpu and no change in susceptibility to HIV-1 Vpu (figure 4B), thereby demonstrating that these two amino-acids play no role in species-specific regulation of human tetherin and *C. denti* tetherin by HIV-1 and SIVden Vpu proteins [3], [27], [30], [31].

**Figure 5:**
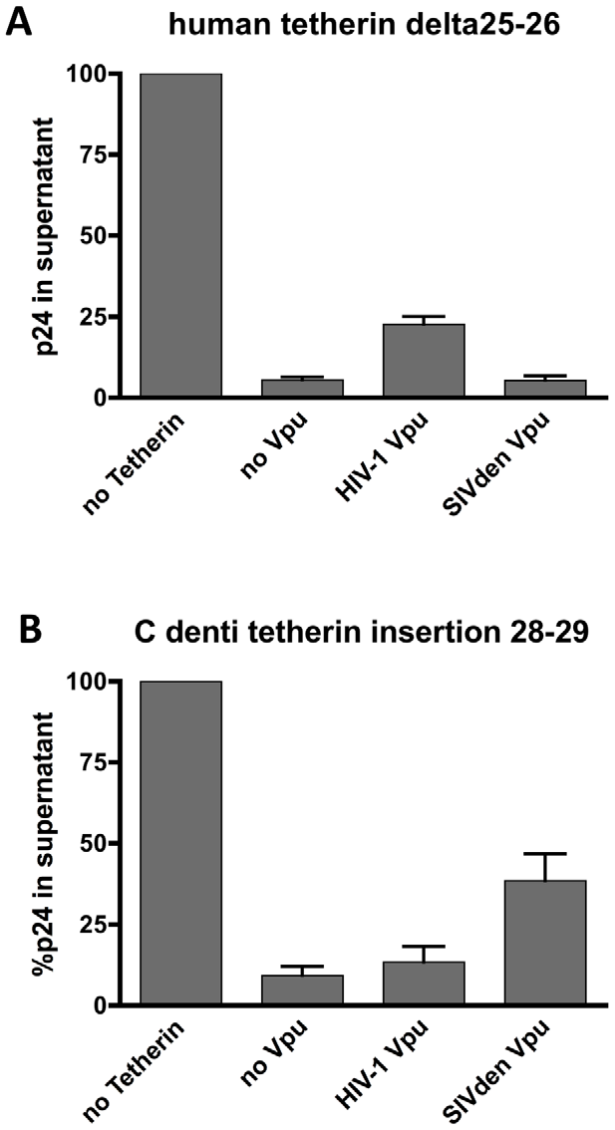
Identification of the residues responsible for the species-specific effect of HIV-1 Vpu and SIVden Vpu. Amino acid substitutions were introduced into full-length human tetherin: (A) P40L, (B) T45I, (C) P40L+T45I. HIV-1 Vpu and SIVden Vpu were tested for their ability to rescue HIV-1ΔVpuΔNef virus release in cells expressing the different human tetherin mutants. Transfection and assay conditions were the same as described in Figure 4 legend.

We then focused our study on the residue 40P/43L/43P (human/*C. denti*/*C. neglectus* numbering respectively) because of the divergence between *C. denti* and *C. neglectus* and on residue 45T/48I/48I that was shown to be required for the antagonism by Vpu [27], [31], [32]. To evaluate the role of these residues, individual and combined substitutions were introduced in human tetherin. As shown on figure 5 A and B, neither of the P40L or T45I substitutions alone were able to alter the strong specificity of HIV-1 Vpu for human tetherin, even if the P40L mutation significantly increased susceptibility of human tetherin to SIVden Vpu. When both P40L and T45I were expressed together in human tetherin, however, susceptibility of this mutant protein to HIV-1 Vpu dropped sharply, while susceptibility to SIVden Vpu increased to 50%, a level close to that observed with wild-type C. denti tetherin. In contrast, reverse substitutions at these positions in C. denti tetherin failed to produce a similar clear-cut change in species specificity (data not shown).

Overall, these findings emphasize that in spite of numerous differences in the transmembrane region of human tetherin and *C. denti* tetherin, most of the species-specificity of their susceptibility by the two different Vpu proteins tested here are essentially carried by the combined residues 40 and 45 in human tetherin.

### Species-specificity of tetherin antagonism by HIV-1, SIVden and SIVdeb Nef

**Figure 6:**
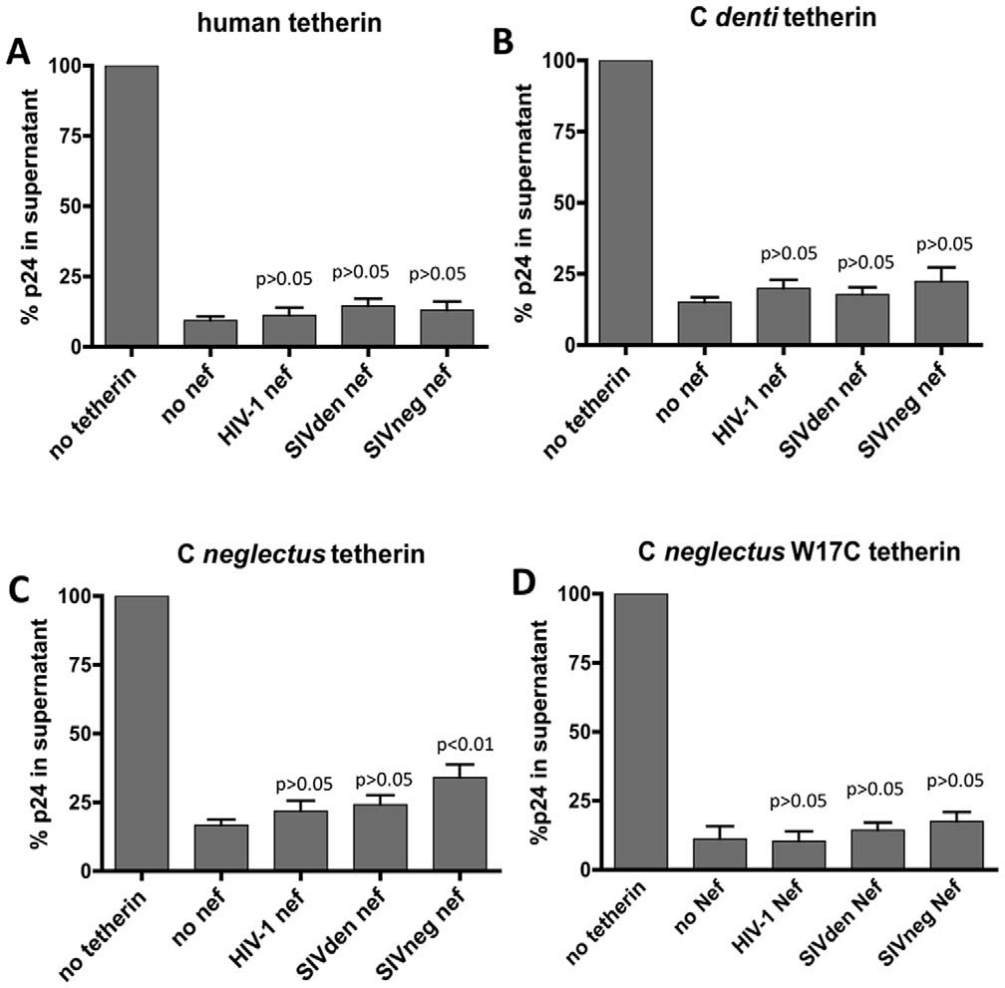
Tetherin antagonism by different Nef proteins. HIV-1 Nef, *C. denti* Nef and *C. neglectus* Nef were tested for the ability to rescue virus release *using vpu* and *nef*-deficient strain of HIV-1 in the presence of human (A), *C. denti* (B), *C. neglectus* (C) and *C. neglectus* (W17C) (D) tetherin. The p24 antigen yield was measured from 293T cells cotransfected with a HIV-1ΔVpuΔNef proviral construct (500 ng), with pcDNA3 vectors expressing the indicated tetherin protein and in combination with plasmids expressing different Nef proteins. Twenty-four h post-transfection, the amount of virus released into the cell culture supernatant was measured by HIV-1 p24 antigen-capture ELISA. All values are shown relative to those obtained in the absence of tetherin expression vector (100%). The indicated p values correspond to the comparison with the «no vpu» sample.

In addition to evaluate species-specificity of human, *C. denti* and *C. neglectus* tetherin to different Vpu proteins, we also examined the extent to which these tetherins were affected by the Nef proteins from HIV-1, SIVden and SIVdeb. As shown on figure 6A, the antiviral effect of human tetherin was not affected by any of the three Nef proteins, consistent with the fact that human tetherin carries a 5 amino acid deletion in its cytoplasmic domain that makes it insensitive to down-regulation by Nef [3], [16]. None of the three Nef proteins expressed any significant effect on *C. denti* tetherin (figure 6B), despite that this tetherin displays no deletion in its cytoplasmic domain. The only tetherin for which some effect of Nef could be measured was *C. neglectus* tetherin (Figure 6C): a small, albeit significant, antagonistic effect of this tetherin was seen with the Nef protein from SIVdeb, the virus that naturally infects *C. neglectus* monkeys. Interestingly, substitution of tryptophan at position 17 in *C. neglectus* tetherin by a cysteine, as found in *C. denti* tetherin, reduces the extent to which SIVdeb Nef counteracts BST2 activity on virion release (Figure 6D), suggesting that this residue participates to the species-specific activity of Nef. Thus, although clearly not as marked as in the case of Vpu, some extent of species-specificity can be seen in the counteracting effect of Nef on the tetherin proteins from the species studied here. Interestingly, the only antagonistic Nef protein was from the virus that does not express a Vpu protein, an effect that was only found significant when targeting the tetherin protein from its natural host species.

### Effect of SIVden and HIV-1 Vpu proteins on human and *C. denti* tetherin expression profiles

**Figure 7:**
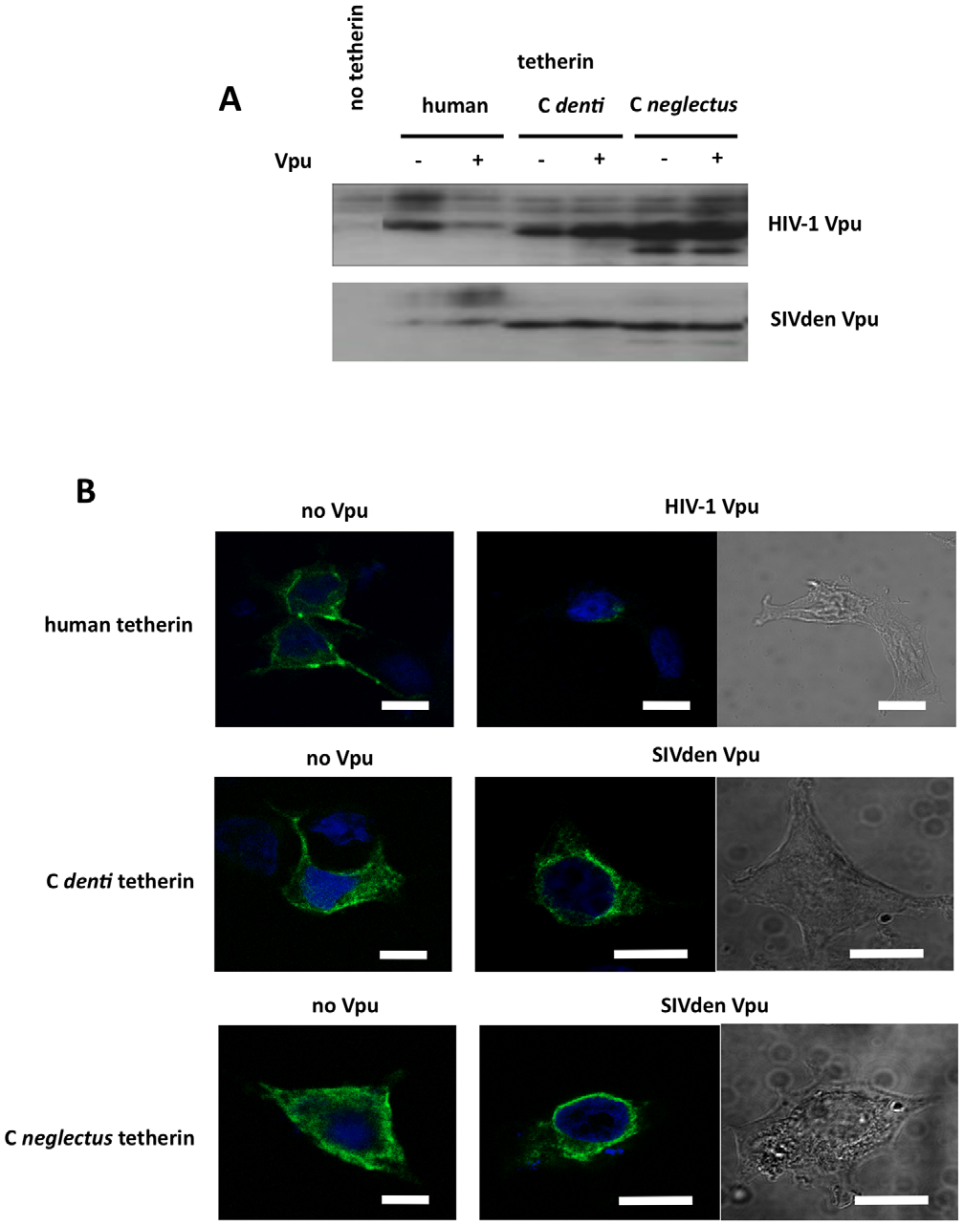
SIV Vpu does not reduce total tetherin levels but leads to depletion of tetherin from the cell surface. (A) 293T cells were cotransfected with HIV-1ΔVpuΔNef proviral construct (500 ng) and a plasmid encoding HA-tagged tetherins (human (100 ng), *C. denti* (1000 ng) and *C. neglectus* (1000 ng) and with or without HIV-1 Vpu plasmid (250 ng) or SIVden Vpu plasmid (250 ng). The effect of the two Vpu constructs on tetherin protein levels was monitored by Western blotting, using an anti-HA antibody. Tetherin migrated as several species in SDS-PAGE analyses, presumably as a result of heterogeneus glycosylation. The depicted gel is representative of three independent experiments. (B) Subcellular localization of tetherin. 293T cells were cotransfected with 1000 ng of DNA encoding the indicated tetherin proteins (HA-tagged), with a proviral construct (1000 ng) and with a HIV-1 Vpu or SIV Vpu plasmid (500 ng). 24 h after transfection the cells were fixed, permeabilized, and stained with rat anti-HA (green) monoclonal antibody (clone 3F10, Roche Applied Science). Nuclei are stained with DAPI (blue). Cells were examined by confocal microscopy. Images are representative of three independent experiments. Scale bars represent 10 µm.

We next examined the mechanisms through which Vpu proteins antagonized the antiviral effects of the tetherin proteins studied here. Two mechanisms have been proposed to explain down-regulation of tetherin by Vpu: accelerated degradation and redistribution, which results in reduced surface expression but conserved overall amounts of tetherin protein. Each of the three tetherin expression plasmids was co-transfected in 293T cells in the presence or absence of the HIV-1 or SIVden Vpu expression plasmids. The transfected cells were subjected to western blot analysis of tetherin content using an anti-HA antibody. As shown on figure 7A, HIV-1 Vpu had a marked effect on total amounts of human tetherin. In contrast, SIVden Vpu, which exerted a strong antagonistic effect on the antiviral properties of *C. denti* tetherin, did not produce any change in the total amounts of this protein. As expected from the results of our experiments evaluating antagonism of tetherin antiviral activity, HIV-1 Vpu had no effect on *C. denti* tetherin and *C. neglectus* tetherin expression levels. Similarly, SIVden Vpu did not reduce cellular amounts of human tetherin and *C. neglectus* tetherin.

To further address the possibility that HIV-1 and SIVden Vpu proteins exerted their antagonistic effects on tetherin through different mechanisms, we used confocal microscopy to examine the cellular distribution of tetherin in the absence and presence of Vpu proteins. Representative images are shown on figure 7B, and a quantitative analysis, based on random examination of 100 cells in each of the transfection conditions, is presented on Table 1. In the absence of Vpu, human tetherin, *C. denti* tetherin and *C. neglectus* tetherin are all localized both at the cell surface and in intracellular compartments. In the presence of HIV-1 Vpu, the pattern of expression of human tetherin changed dramatically. In most cells, the amount of human tetherin expressed became very weak and diffuse, while in ∼10% of cells tetherin expression was restricted to the perinuclear region. A different pattern was seen with *C. denti* tetherin in the presence of SIVden Vpu. In this case, only redistribution was observed and tetherin was expressed in cytoplasmic and perinuclear compartments, at the expense of surface expression. This is fully consistent with the notable impact of SIVden Vpu protein on *C. denti* tetherin antiviral activity in spite of the lack of effect of SIVden Vpu on overall expression of *C. denti* tetherin seen on western blots.

**Table 1:** Localization of human and primate tetherins in presence or absence of Vpus.

expressed proteins	surface+cytoplasm *	perinuclear *	diffuse very weak *	P (chi-square)
human tetherin	87	13	-	
human tetherin+HIV-1 Vpu	11	12	77	<0.001
C *denti* tetherin	89	11	-	
C *denti* tetherin+SIVden Vpu	30	70	-	<0.001
C *neglectus* tetherin	90	10	-	
C *neglectus* tetherin+SIVden Vpu	61	39	-	<0.001

* percentage of cells expresssing tetherin following the indicated pattern.

## Discussion

Primate lentiviruses have evolved three distinct mechanisms to counteract the effects of tetherin, a restriction factor that retains virions at the surface of the producer cell, thereby limiting their ability to spread to new target cells. The majority of these viruses takes advantage of the multifunctional Nef protein, a protein that, depending on the virus, also has the ability to downmodulate surface expression of a variety of host cell membrane proteins such as CD3, CD4 or MHC class I [6], [33]. A subset of *Cercopithecus* lentiviruses have acquired a *vpu* gene [24], [34], [35], that has the capacity to interact with the transmembrane region of tetherin, an interaction that leads to endocytosis and/or accelerated degradation of the restriction factor [6], [17], [18]. Of note, the Vpu proteins of some primate lentiviruses also exert an inhibitory effect on surface expression of CD4 [6], [36]. Finally, in a few viruses for which neither Vpu nor Nef seem able to counteract tetherin, this function is achieved by the intracellular domain of their Env protein [28], [37], [38].

The evolutionary mechanisms explaining why some primate viruses rely on Vpu for regulation of tetherin while still expressing a Nef protein are not fully explained. Differents parameters seems to have driven evolution towards the use of Nef or Vpu. In SIVcpz, a virus infecting chimpanzees which was reported to arise from recent recombination between SIVgsn (a Vpu-expressing monkey virus) and SIVrcm (a monkey virus without a vpu gene) [39], the SIVgsn-derived Vpu protein fails to efficiently counteract chimpanzee tetherin but this function is fulfilled by SIVrcm-derived Nef [6], [17], [18]. In humans, a different situation is encountered: the human tetherin protein carries a deletion of a 5-amino acid motif in its cytoplasmic domain making it insensitive to Nef, a situation that has probably forced (at least in HIV-1 group M) rapid evolution of a Vpu protein with strong activity against human tetherin [3], [6], [16], [17], [18], [19]. Yet another particular situation is encountered with HIV-2 in humans, as HIV-2 is derived from SIVsmm, a monkey virus with no vpu gene. Because it could neither rely on Nef nor on Vpu for down-regulation of human tetherin, HIV-2 has evolved an Env protein that has acquired this property [38], [40]. Similarly, SIV from Tantalus monkeys (SIVtan) encodes an envelope glycoprotein able to counteract tetherin from Tantalus monkeys, rhesus monkeys, sooty mangabeys, and humans [28].

These observations and assumptions, however, do not fully explain why, earlier in primate lentiviral evolution, some monkey viruses have acquired a Vpu protein while others have solely relied on their Nef protein for counteracting tetherin.

The ability of SIVden Vpu protein to antagonize tetherins was already addressed [6], [18]. In this study we have examined the anti-tetherin activity of two closely related monkey viruses, SIVden from *C. denti*, which expresses a Vpu protein, and SIVdeb from *C. neglectus*, which does not have a Vpu gene. The tetherin proteins from these two monkey species are closely related, diverging only by a few polymorphisms in their cellular and transmembrane domains, which are known to determine tetherin sensitivity to Vpu and Nef, respectively.

We first compared the sensitivity of human, *C. denti* and *C. neglectus* tetherins to SIVden Vpu and HIV-1 Vpu (Figure 2). HIV-1 and SIVden Vpu proteins strongly and almost exclusively affected the antiviral activity of human and C. denti tetherins, respectively. Interestingly, *C. neglectus* tetherin appeared more sensitive to SIVden Vpu than to HIV-1 Vpu, consistent with the high homology found between the Vpu-sensitive domains of *C. denti* and *C. neglectus* tetherins.

In our study, the specific activity of SIVden Vpu on *C. denti* tetherin was explained by the presence in tetherin of a leucine at position 40 and of an isoleucine at position 45 fond in *C. denti* tetherin. Introduction of these two amino acid simultaneously in human tetherin reproduced the phenotype observed with wild-type *C. denti* tetherin (Figure 3).

The deletion of two amino acids at position 25–26 in human tetherin, as well as the insertion of the corresponding amino acids in *C. denti* tetherin, however, failed to exert any effect on virus-host specificity (Figure 4).

In this study, we also examined susceptibility of the three tetherins to the Nef proteins from HIV-1, SIVden and SIVdeb. Consistent with previous studies, the antiviral activity of human tetherin was resistant to all three Nef proteins. Clearly, *C. neglectus* tetherin was the most sensitive to Nef, an effect that was most significant using SIVdeb Nef, a finding fully consistent with the fact that this SIVdeb does not express a Vpu protein and appears to rely on Nef for counteracting tetherin. Interestingly, introduction of the W17C substitution in *C. neglectus* tetherin, which reproduces the sequence of *C. denti* tetherin at this site, abrogated the effect of Nef, emphasizing the importance of this residue in virus-host adaptation. It is noteworthy that the effect of Nef on *C. neglectus* tetherin, although significant, was only partial (30% rescue of the block in virus release exerted by tetherin), and reminiscent of observations by Lim et al. with SIVagm from African green monkeys [3]. As suggested by these authors for SIVagm, it is possible that in *C. neglectus*, infection by SIVdeb may induce only limited induction of interferon type I production, thereby limiting the amount of tetherin to which the virus is confronted during *in vivo* replication. Similarly, the impact of SIVden Vpu on *C. denti* tetherin is clearly partial (60%) compared to that observed with HIV-1 and human tetherin. Again, such partial activity is suggestive of the fact that *C. denti* may not need as much neutralization of tetherin as that required for human infection by HIV-1. Nonetheless, the fact that SIVden relies essentially on Vpu for activity on tetherin, and the fact that this effect on *C. denti* tetherin is clearly stronger than that of SIVdeb Nef on *C. neglectus* tetherin, together suggest that during infection of *C. denti*, SIVden may be confronted to higher levels of tetherin expression than those in SIVdeb-infected *C. neglectus* monkeys.

These results do not exclude the possibility that the Env protein from SIVdeb may also participate in antagonizing the antiviral activity of tetherin, as has been observed for HIV-2 [38], [40] and SIVtan [28], although viruses that employ two different proteins to inhibit tetherins have not as yet been described. Testing this possibility will require the identification of functional SIVdeb Env proteins.

Another interesting finding of our study, pertaining to the particularly strong activity of HIV-1 Vpu on human tetherin, was the striking difference observed on the pattern of cellular expression of the three tetherins tested here in response to the two Vpu proteins. As shown on figure 5 and table 1, HIV-1 Vpu almost completely abolished expression of human tetherin, confirming earlier findings that human tetherin was not only removed from the cell surface but also directed to an efficient degradation pathway. In contrast, the activity of SIVden Vpu on both *C. denti* and *C. neglectus* tetherins, only resulted in redistribution of these proteins, with a predominantly perinuclear expression. Although it is conceivable that this effect is related to differences in intrinsic recycling and degradation pathway targeting properties between HIV-1 and SIVden Vpu proteins, it is also possible that redistribution and perinuclear expression without degradation is related to the partial activity of SIVden Vpu on *C. denti* and *C. neglectus* tetherins. In this case the incomplete activity of SIVden Vpu would be sufficient for redirecting tetherins to the perinuclear compartment but not quite sufficient for achieving full targeting to a degradation compartment.

Our findings endorse the assumption that Vpu and Nef-mediated tetherin antagonism are original properties of primate lentiviruses, and the resistance of human tetherin to primate lentivirus Vpus and Nefs seems to represent a significant barrier to zoonotic transmissions [6], [16], [17], [18], [19].

## Materials and Methods

### Construction of the plasmid HIV-1ΔVpuΔNef-Renilla

The Vpu region of the plasmid deleted of Nef pNL4-3-lucR [41] was deleted and replaced by the deletion mutant produced by Klimkait et al (1990). This mutation comprises a deletion of 48 nucleotides immediately following the initiator methionine codon of the vpu ORF plus a 7-nucleotide insertion, and results in a minus-1 frameshift and premature termination after 14 missense codons [42].

### Tetherin cloning

Tetherin genomic regions were PCR amplified from HeLa cells and from *C. denti* and *C. neglectus* uncultured peripheral blood mononuclear cell (PBMC) DNA. A unique forward primer was used (5′-TGGAACTTGCTATTGGTCAG-3′). Two reverse primers were synthesized, one for human and *C. denti* tetherins (5′-CCTTCTCACTGGATTCTCC-3′) and one for *C. neglectus* (5′-CCTTCTCACTGGATTCTCA-3′).

Splice-overlap-extension PCR was used to splice the introns. The integrity of all PCR derived cDNAs was confirmed by sequence analysis.

N-terminal HA-tagged tetherins were obtained by PCR using a N-terminal primer containing the HA sequence. These products were cloned into the KpnI-Xho1 sites of pcDNA3.1/CT-GFP-TOPO (Invitrogen).

### Vpu and Nef cloning

The pNG227 plasmid containing four copies of Mason-Pfizer monkey virus constitutive transport element was a gift of Michael H. Malim. The two Vpu proteins and the three Nef proteins were cloned in pNG227 using the XbaI-SalI sites.

Tetherin-HA for confocal microscopy was obtained by inserting a single HA epitope tag to the ectodomain of the three BST2 proteins by primer-directed mutagenesis using PCR [27]. Overlap-extension PCR approaches were used to insert the HA-tag at a position 463 nucleotide of human tetherin protein, using PCR-A primers 5′-CCGGTACCATGGCATCTACTTCGTATGA-3′ (forward) and 5′-AGTCTGGGACGTCGTATGGGTATGCGGCGTAGTACTTCTTGTCCGCGATTCTCAC –3′ (reverse), and PCR-B primers 5′- CATACCCATACGACGTCCCAGACTACGCTGCCCCCAGCTCCCAGGACTCCAGCTC –3′ (forward) and 5′- GTCTCGAGTCACTGCAGCAGAGCGCTTAGG –3′ (reverse). For the monkey tetherins, the HA epitope was inserted at a position orthologous to nucleotide 463 using primers *C. denti* tetherin PCR-A 5′- CCGGTACCATGGCACCTATTTTGTATGACT -3′ (forward), 5′- GGCAGCGTAGTCTGGGACGTCGTATGGGTATGCGGCGGAGTTCGTGTCCGCGATT-3′ (reverse), PCR-B 5′- TACCCATACGACGTCCCAGACTACGCTGCCGCCAGCCCCCAGGACTCCAGCTGCG-3′ (forward), and 5′- GGCTCGAGTCACAGCAGCAGAGCGCCCAAG-3′ (reverse), respectively. *C. neglectus* tetherin primers PCR-A 5′- CCGGTACCATGGCACCTATTTTGTATGACT-3′ (forward), 5′- GCGTAGTCTGGGACGTCGTATGGGTATGCGGCGGAGTTCGTGTCCGCGATTCTCA-3′ (reverse), PCR-B 5′- TACCCATACGACGTCCCAGACTACGCTGCCAGCCCCCAGGACTCCAGCTGCGCGG-3′ (forward), and 5′- GTCTCGAGTCACAGCAGCAGAGCGCCCAAG-3′ (reverse), respectively. PCR fragments were cloned into pcDNA3 vector by inserting a KpnI-XhoI restriction site. All plasmids were confirmed by nucleotide sequence analyses.

### Cell lines

The human cell line 293T were obtained from the American Type Culture Collection (ATCC Cat No. CRL-11268) and cultured according to the instructions provided.

### Virus yield and tetherin antagonism

To determine the capability of Vpu and Nef to antagonize tetherin, 293T cells were seeded in a 12-well plate and transfected with 500 ng of the pNL4.3-ΔVpuΔNef proviral construct, tetherin expression vectors [human (100 ng), *C. denti* (1000 ng) and *C. neglectus* (1000 ng)], 250 ng µg HIV-1 Vpu or SIVden Vpu expression plasmids and 600 ng of Nef expressing plasmids. A pcDNA3 vector (Invitrogen) was used to equalize the DNA concentration. Transfection were made using jetPEI transfection reagent (PolyPlus transfection) according to manufacturer's recommendations. At 42 hours post-transfection supernatants were harvested, clarified by centrifugation at 800 g and analyzed for p24 antigen by an enzyme-linked immunosorbent assay (ELISA) kit (Innogenetics, Belgium).

### Western blot

To monitor tetherin expression, 293T cells were cotransfected with the indicated amount of N-terminally HA-tagged tetherin expression constructs [(human (100 ng), *C. denti* (1000 ng) and *C. neglectus* (1000 ng)], proviral constructs pNL4.3-ΔVpuΔNef-Renilla (500 ng) and HIV-1 Vpu (250 ng) or SIVden Vpu (250 ng) plasmids. The transfections were made as described above. One day post-transfection cells were washed once with PBS and lysed in the sample buffer [50 mM Tris-HCl (pH 6.8), 2% SDS, 100 mM DTT, 10% glycerol and 0,005% bromophenol blue]. Proteins were solubilized by heating for 10 min at 95°C. Residual insoluble material was removed through centrifugation (10 min at 11000× g). Cell lysates were separated on SDS-PAGE and transferred to Nitrocellulose membranes and probed with anti-HA Rat antibody (Roche, 3F10, 1∶1000 dilution). Specific antibody-antigen complexes were identified using horseradish peroxidase-labeled goat anti-rat IgG secondary antibodies (AP136P, Millipore) and then incubated with the chemiluminescent substrate (RPN2135, Amersham). Densitometry quantification was achieved using ImageJ software.

For internal controls, blots were re-incubated with mouse antibodies specific for HIV1 p24 (ab9071, Abcam) and actin (A 2066, Sigma). Subsequently, blots were probed with anti-mouse or anti-rabbit IRDye antibodies (Odyssey 926-32222, 926-32213) and proteins revealed using a LI-COR Odyssey scanner.

### Confocal microscopy

Subconfluent monolayer of adherent human 293T cells grown on coverslips in 6-well plates were cotransfected with tetherin plasmids carrying HA-tag in the ectodomain (1000 ng), with a proviral constructs pNL4.3-ΔVpuΔNef (1000 ng) and HIV-1 Vpu or SIVden Vpu (500 ng), and with appropriate amounts of the empty pcDNA3 vector used as carrier plasmid DNA to normalize the quantity of DNA transfected per well. The transfections were made as described above. After 24 hours, cells were fixed with 3% formaldehyde in PBS for 15 min at room temperature, washed three times with 50 mM ammonium chloride in PBS. The cells were incubated with 1% bovine serum albumin in PBS. For labeling the cell surface, the cells were incubated with anti-HA rat primary antibodies (Roche, 3F10, 1∶2000 dilution) for 1 h. For permeabilization, cells were treated for 5 min at room temperature with 0.1% Triton X-100 buffered with PBS. After washing three times with PBS, non-specific binding was blocked with 1% bovine serum albumin in PBS for 1 h at room temperature. Cells were then incubated for 1 h at room temperature with an anti-HA Rat primary antibodies (Roche, 3F10, 1∶2000 dilution). Cells were washed for 20 min in PBS, and incubated with anti-mouse immunoglobulin G-Alexa Fluor (IgG-Alexa Fluor 488, 1∶500 dilution) for 1 h at room temperature. Finally, cells were washed again for 20 min in PBS and then mounted using Vectashield+DAPI (Vector Laboratories, Burlingame, CA). Cells were then examined using a LSM ZEISS 510 confocal microscope.

### Nucleotide sequence accession numbers

The sequences have been submitted to the EMBL database: *C. denti* (HE680870), *C. neglectus* (HE680871) tetherins and SIVneg Nef gene (HE680872).

Others accession number are: Nef pNL4.3 (M19921), SIVden Nef (AJ580407) and SIVden Vpu (CAE46404).

### Statistical analysis

All statistical calculations were performed with a one-way analysis of variances (ANOVA) using GraphPad PrismVersion 4.0. Post test comparisons, performed only if p<0.05, were made using Bonferroni's Multiple Comparison Test.
